# Overcrowding and health in two impoverished suburbs of Johannesburg, South Africa

**DOI:** 10.1186/s12889-019-7665-5

**Published:** 2019-10-24

**Authors:** Vusumuzi Nkosi, Tanya Haman, Nisha Naicker, Angela Mathee

**Affiliations:** 1 Environment and Health Research Unit, South African Medical Research Council, Johannesburg, 2094 South Africa; 2 School of Health Systems and Public Health, Faculty of Health Sciences, University of Pretoria, Pretoria, 0001 South Africa; 3 Department of Environmental Health, Faculty of Health Sciences, University of Johannesburg, Cnr Sherwell and Beit Street, Health Clinic building, 2nd Floor, Doornfontein Campus, Doornfontein, Johannesburg, 2094 South Africa; 4 School of Public Health, Faculty of Health Sciences, University of the Witwatersrand, Johannesburg, 2094 South Africa; 5 National Institute for Occupational Health, National Health Laboratory Services, Johannesburg, 2094 South Africa

**Keywords:** Overcrowding, Health, South Africa, Housing, Environmental health

## Abstract

**Background:**

Rapid urbanization, unmatched by an associated supply of housing, has resulted in overcrowding in the cities of many developing countries, including in Johannesburg, South Africa. Household overcrowding has been associated with a range of ill-health outcomes, including acute respiratory infections and diarrhoeal diseases. The aim of this study was to describe the levels of household crowding, and examine associations with respiratory and gastrointestinal symptoms in selected two low-income neighbourhoods in Johannesburg.

**Methods:**

Questionnaire data from a panel study conducted over an 11-year period between 2006 and 2016 were extracted to conduct the analyses. Structured questionnaires, designed to collect information on housing conditions, socio-economic and health status were administered to adult representatives of households occupying the primary dwelling on pre-selected study sites.

**Results:**

Over the 11-year study period, levels of overcrowding remained unchanged. Around 57.6% of dwellings in the study neighbourhoods were determined to be overcrowded in relation to international guidelines. Results from the multiple logistic regression analyses indicated that crowded dwellings were associated with elevated levels of acute respiratory and gastrointestinal symptoms, as well as fever/chills.

**Conclusion:**

Respondent perceptions varied from objective measures of overcrowding. Crowded dwellings were associated with elevated reports of acute respiratory and gastrointestinal symptoms, as well as fever/chills.

## Background

Housing is well established to be a setting in which a complex and inter-related network of physical, social, economic and behavioral factors interplay to impact on the health of occupants [1]. Sufficient space to support the maintenance of clean indoor air, reduce the risk of disease transmission, reduce the risk of noise exposure and meet needs for privacy, amongst other factors, is a fundamental element of healthy housing. It is challenging to discern the health impacts of a single aspect of housing, such as overcrowding, from the range of other potential housing elements that may impact on health, such as dampness, type of fuel used for daily cooking, water quality, smoking practices. However, a range of controlled studies conducted across the globe have shown significant associations between overcrowded housing and, for example, tuberculosis [2], hospitalization for influenza [3], pneumonia and other acute respiratory infections [4, 5], meningococcal disease [6] and rheumatic fever [7].

The world population has increased significantly over the past century and is predicted to reach 9.7 billion by 2050, with developing countries, especially the African region, being amongst the worst affected [8]. Overcrowding is already particularly severe in developing countries, and alongside a process of urbanization, is expected to increase and intensify further in years to come. In China, for example, a study undertaken in 2016 showed that 47.5% of urban households lived under overcrowded (overcrowding was defined as more than two people per habitable room) housing conditions [9]. Similarly, a study undertaken in Nigeria in 2015 showed that on average, households comprising between 8 and 12 persons lived in dwellings with only two rooms [10]. In Ghana, 44.5% of households are estimated to live in overcrowded housing [11].

South African cities such as Johannesburg, being a magnet for people from the hinterland as well as beyond national borders, seeking work, educational, recreational and other opportunities, have relatively high (and increasing) population densities, and have been particularly prone to overcrowding. Research covering the period between 2002 and 2014 showed that nation-wide, more than one-quarter of formal, low-cost dwellings in South Africa were overcrowded [12]. Socio-economic status, gender and population group count amongst a range of risk factors recognized to be associated with household overcrowding [13–15]. In the aftermath of colonization and apartheid in South Africa, poverty, and the associated overcrowding, remains inequitably distributed across the population. The Black African and Coloured population are worst affected, with research undertaken in 1995 showing that 66% of Black African and 59% of Coloured groups lived in overcrowded conditions. This is in sharp contrast to 11% in the White population being similarly affected [16].

There is a paucity of detailed, neighbourhood level research on the levels of overcrowding in various South African communities. Information on the implications of overcrowded living conditions for health is even more sparse. This study investigated levels of overcrowding in two neighbourhoods in Johannesburg, as well as the associations with selected ill-health symptoms.

## Methods

### Sampling and data collection

Data for this paper were extracted from the database of an 11-year panel study undertaken in two suburbs in Johannesburg between 2006 and 2016 [17]. Surveys were conducted annually during winter at dwellings that had been randomly selected at the time of commencement of the study in 2006. Data on socio-economic factors, living conditions and health status were collected through the administration of pre-structured questionnaires by trained field workers to a household respondent of at least 18 years of age. Information was collected only for the primary (main) household on each study site, with households living in secondary or backyard dwellings being excluded. Households were defined as a group of people eating meals together. The study sites were Braamfischerville and Riverlea, which were constructed around 1996 and 1961 respectively. Both Braamfischerville and Riverlea are large-scale, low-cost housing developments, with brick and mortar dwellings of around 30 m^2^. Most dwellings comprise a kitchen, living room (in an open-plan format in Braamfischerville, but separated in Riverlea), bathroom and two or three bedrooms [18]. In general, Braamfischerville and Riverlea dwellings have corrugated metal sheeting and asbestos roofs, respectively.

The targeted number of dwellings/households for inclusion in the study was 200 in each site. The average response rate over the 11-year period of study was 67.2%. The same dwellings were visited each year to collect information on the current resident households; thus in some cases, information may have been collected on the same household over the 11-year study period or part thereof, while at other dwellings information may have been collected on several different households over the same period.

There is a range of definitions of overcrowding across international institutions and countries. According to the United States Department of Housing and Urban Development, there are four key measures that are used to define overcrowding, namely number of persons-per-room, persons-per-bedroom, unit square footage-per-person and persons-per-room by unit square foot-per-person. Persons-per-room is the most utilized measure to quantify overcrowding [19]. United Nations-HABITAT (UN-HABITAT), defines overcrowding as more than two people per room (excluding bathrooms but including kitchens and living rooms) [20], while according to the World Health Organization overcrowding can be measured as the average living area per person in the place of residence [21]. According to the Environmental Health Indicators New Zeland, overcrowding is defined as needing one or more bedrooms and severe overcrowding as needing two or more bedrooms [22]. For the purpose of this paper, the UN-HABITAT definition of residential overcrowding as more than two people per room (excluding bathrooms but including kitchens and living rooms) [20] was adopted. Overcrowding was further classified into three categories: none, moderate and extreme overcrowding. In terms of the UN-Habitat definition, dwellings were defined as moderately overcrowded if occupied by > 2 but < 5 people per room, whereas extreme overcrowding was defined as a dwelling with > 6 people per room [19]. In order to obtain relevant health information, respondents, aged at least 18 years, were asked whether any member of the household had experienced a selection of respiratory and gastrointestinal symptoms, using a two-week recall period.

### Statistical analyses

Respondents were asked about the number of people living in the dwelling, the number of rooms in the dwelling, socio-demographic profile, period of residence in the current dwelling, educational status of the head of the household, smoking practices of household members, whether the household included children under the age of 5 years and experience of selected respiratory and gastrointestinal ill-health symptoms. Data were entered into MOBENZI (mobile data collection and capturing tool) and exported into STATA statistical package version 14 (StataCorp. 2015. Stata Statistical Software: Release 14. College Station, TX: StataCorp LP) for analyses. The prevalence of the health measures was calculated by dividing the number of households for whom health symptoms were confirmed by the number of households participating in the study for a particular site. A chi-square test was applied, to determine the relationship between the perceived overcrowding in own dwelling, suburb and degree of overcrowding in own dwelling as defined by the UN-HABITAT. Data for each site were weighted and the STATA survey command used to take account of clustering effects and the study design. Trend analysis was calculated using the np trend command. Crude and adjusted odds ratios (OR) and 95% confidence intervals (CI) were calculated using univariate and multiple logistic regression analysis (LRA) to estimate the likelihood of having acute respiratory and gastrointestinal symptoms, as well as fever/chills. To obtain adjusted ORs for the effect of overcrowding on the health outcomes were placed in the initial LRA model. This was followed by the addition of a potential confounder in a stepwise manner starting with the most statistically significant from the univariate analysis. Possible confounders included the following: sex, study area, head of the household education level, whether any member of the household smoke at home, household total monthly income, having children under the age of five in the household and period of residence in the current dwelling. Each time a new potential confounder was added to the model if the effect estimate between the overcrowding and health outcome of interest already in the model changed by 5%, the additional variable was retained in the final multiple LRA, otherwise, the variable was removed and a different one was added [23]. The most parsimonious multiple LRA models were reported [24].

## Results

### Study population

Table 1 shows the profile of the population in the study. Data from 3121 interviews conducted over the 11-year study period, were analysed; there were more female (2017, 64.6%) than male (1104, 35.4%) respondents. The majority (1884, 59.2%) of the heads of households had achieved a high school educational qualification. Most households (1589, 50.9%) had a monthly income between R1001 – R5000. More than 50% of respondents reported less than 10 years of duration of residence in their current dwelling.

**Table 1:** Socio-demographic profile of the study site (combined 11 years data)

Characteristics	*N* = 3121
	n (%)
Sex (head of household)	
Female	2017 (64.6)
Male	1104 (35.4)
Level of education (head of household)	
None	186 (6.0)
Primary	773 (24.8)
Secondary	1844 (59.2)
Tertiary	294 (9.4)
Missing	20 (0.6)
Household total monthly income	
No income	765 (24.5)
R1001 – R5000	1589 (50.9)
R5001 – R 10000	346 (11.1)
> R10000	246 (7.9)
Missing	175 (5.6)
Any member of the household smokes at home	
Yes	1082 (34.7)
No	2039 (65.3)
Households with children under 5 years of age	
Yes	1356 (43.4)
No	1765 (56.6)
Period of residence in the current dwelling	
< 10 years	1660 (53.2)
> 10 years	1461 (46.8)
Perception of overcrowding in own dwelling	
No problem	2352 (75.4)
Moderate problem	373 (12.0)
Major problem	395 (12.7)
Missing	1 (0.03)
Perception of overcrowding in the suburb	
No problem	1509 (48.4)
Moderate problem	620 (19.9)
Major problem	992 (31.8)
Degree of overcrowding in own dwelling by UN HABITAT definition	
Not overcrowded^a^	1322 (42.4)
Moderately overcrowded^b^	1518 (48.6)
Extremely overcrowded^c^	281 (9.0)

figures in parentheses are percentages

^a^≤ two people/room

^b^more than 2 but ≤5 people/room

^c^more than 6 people/room

### Acute respiratory and gastrointestinal symptoms, fever/chills prevalence

The prevalence of acute respiratory and chills among/chills, for the total sample, are shown in Table 2. Prevalence of acute respiratory symptoms and fever/chills were higher compared to those of gastrointestinal symptoms.

**Table 2:** Prevalence of acute respiratory, gastrointestinal symptoms and fever/chills

Outcome	Response Item	Number of partcipants (n)	Percentage of participants
Acute respiratory symptoms			
Wet cough	Yes	291	9.3
	No	2830	90.7
	Total	3121	100
Dry cough	Yes	287	9.2
	No	2834	90.8
	Total	3121	100
Runny/blocked nose	Yes	387	12.4
	No	2734	87.6
	Total	3121	100
Gastrointestinal symptoms			
Diarrhoea	Yes	76	2.4
	No	3045	97.6
	Total	3121	100
Vomiting	Yes	88	2.8
	No	3033	97.2
	Total	3121	100
Fever/chills			
Fever/chills	Yes	335	10.7
	No	2786	89.3
	Total	3121	100

### Overcrowding: by definition and perception and trends

Respondents’ perceptions of overcrowding within their own dwellings and suburb were poorly correlated with the objective UN-HABITAT measure (*p* < 0.001), (Tables 3 and 4). For example, respondents perceived their dwellings as not overcrowded; whereas their dwellings were moderately overcrowded according to the UN-HABITAT measure. The trend analysis showed no significant change in the level of overcrowding in the two suburbs over the 11-year study period (*p* = 0.12).

**Table 3:** Overcrowding by UN-HABITAT definition and perception in own dwelling

Degree of overcrowding in own dwelling by UN-HABITAT definition				
	Not overcrowded^a^	Moderately overcrowded^b^	Extremely Overcrowded^c^	^#^*P*-Value
Perception of overcrowding in own dwelling				
Major Problem	93 (3.0)	231 (7.4)	71 (2.3)	< 0.001
Moderate problem	111 (3.6)	207 (6.6)	55 (1.8)	
No problem	1118 (35.8)	1079 (34.6)	155 (5.0)	
Total	1322 (42.4)	1518 (48.6)	281 (9.0)	

Figures in parentheses are percentages

^a^≤ two people/room

^b^more than 2 but ≤5 people/room

^c^more than 6 people/room

^#^*P*-value of the chi-square test

**Table 4:** Overcrowding by UN-HABITAT definition and perception in own suburb

Degree of overcrowding in own dwelling by UN-HABITAT definition				
	Not overcrowded^a^	Moderately overcrowded^b^	Extremely Overcrowded^c^	^#^*P*-Value
Perception of overcrowding in own suburb				
Major Problem	392 (12.6)	502 (16.1)	98 (3.1)	0.001
Moderate problem	236 (7.6)	316 (10.1)	68 (2.2)	
No problem	694 (22.2)	700 (22.4)	115 (3.7)	
Total	1322 (42.4)	1518 (48.6)	281 (9.0)	

Figures in parentheses are percentages

^a^≤ two people/room

^b^more than 2 but ≤5 people/room

^c^more than 6 people/room

^#^*P*-value of the chi-square test

### Association between overcrowding and health outcomes

**Fig. 1:**
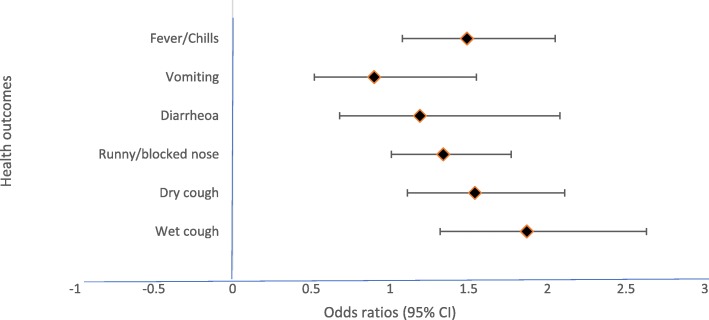
Multiple regression analyses of moderately overcrowded dwellings and health outcomes. Models adjusted for sex, study area, head of the household education level, whether any member of the household smoke at home, household total monthly income, having children under the of five in the household and period of residence in the current dwelling

**Fig. 2.**
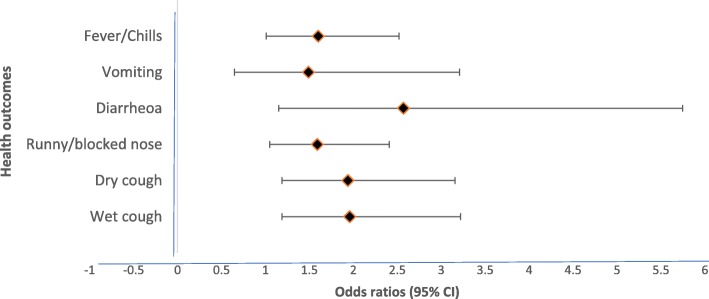
Multiple regression analyses of extremely overcrowded dwellings and health outcomes. Models adjusted for sex, study area, head of the household education level, whether any member of the household smoke at home, household total monthly income, having children under the of five in the household and period of residence in the current dwelling

Univariate analyses of the degree of dwelling overcrowding by UN HABITAT definition, acute respiratory, gastrointestinal symptoms, and fever/chills among the study participants in the study sites are shown in Additional file 1: Table S1. Figure 1 shows the crude odds ratios between levels of overcrowding and health outcomes considered in this study. Results from the multiple logistic regression analysis (LRA) (Fig. 2) indicate that acute respiratory symptoms were statistically significantly associated with moderately, as well as extremely overcrowded dwellings. Diarrhoea was also statistically significantly associated with extremely overcrowded dwellings (OR = 2.57; 95% CI:1.15–5.75). Respondents residing in moderately and extremely overcrowded dwellings were 1.49 and 1.60 times more likely to have fever/chills, respectively. The multiple LRA models were adjusted for the following risk factors: sex, study area, head of the household education level, whether any member of the household smoke at home, household total monthly income, having children under the age of five in the household and period of residence in the current dwelling. The adjusted odds ratios for health outcomes and each risk factor considered in this study are shown in the Additional file 1: Table S2-S6.

## Discussion

The results of the study suggest that a large proportion of dwellings were overcrowded (57.6%) relative to the UN-Habitat definition of overcrowding as more than two people per room (excluding bathrooms but including kitchens and living rooms). The levels determined in this study, are however lower than shown in studies undertaken elsewhere in Africa. In Lagos (Nigeria) for example, around 77% of participants were reported to live in overcrowded dwellings [21]. Also, in these particular sites, over the 11-year period of the study, no statistically significant change in the levels of residential overcrowding was determined. A contributing factor in this regard may be the accelerated housing delivery programmes, such as *Breaking New Ground* that has been underway in South Africa over the past three decades to provide low-cost housing [18].

This study has provided evidence in an urban South African setting of an association between overcrowded housing and ill health, specifically elevated levels of respiratory symptoms and fever/chills in both moderately and extremely crowded houses, as well as diarrhoea in extremely overcrowded housing. The prevalence of diarrhoea for this study was 2.8% and was less than of the research study conducted in low-cost housing settlements of Cape Town in South Africa where a 2-week recall period reported prevalence of diarrhoea was 14%. This difference might be attributed to the fact that the communities that were surveyed in Cape Town were exposed to water with high levels of Ecoli for domestic use [25]. However, the prevalence of vomiting and fever/chills for this study were higher compared to the Cape Town study. Higher prevalence of acute respiratory symptoms for this study was observed compared to the survey conducted in a similarly impoverished suburb of KwaZulu Natal in South Africa [26]. An exchange mechanism for disease transmission in overcrowded housing is through the air by droplets or aerosols when people are sneezing, coughing or talking [27]. Repeated bouts of diarrhoeal or respiratory ill-health may detrimentally affect the quality of life and resistance to other diseases, such as measles and meningitis, as well as incur high costs to both affected individuals and the health services.

A health economics study undertaken at a local hospital in 2010 showed the mean hospital stay for cases of diarrhoeal disease from a rotavirus infection was 4.6 days, and that the cost of in-patient treatment ranged from 5963 to 7256 South African Rands (R). The cost of in-patient treatment adjusted for South African inflation ranged from R9355 to R11383 [633.38 to 770.68 US Dollars (US$)]; the date of the exchange rate used was the 06th September 2019) [28]. Also, the average out-of-pocket expenses for in-patients amounted to approximately R100 (US$6.77; the date of the exchange rate used was the 06th September 2019). The mean annual cost of influenza-associated illness in South Africa was estimated at $270.5 million, 0.08% (5.1% per capita) of the total mean annual gross domestic product [29]. Additional research and full cost accounting may reveal a much higher economic burden to the national budget associated with the prevailing levels of overcrowding in South Africa, especially in the light of the exchange range of diseases that have been associated with overcrowding, including tuberculosis [2], hospitalization for influenza [3], pneumonia and other acute respiratory infections [4, 5], meningococcal disease [6], ear infections [30], skin infections [31] and rheumatic fever [7]. Further detrimental social and environmental consequences of overcrowded housing include exposure to noise, a lack of privacy, mental ill-health [32] and increased violence [33].

In the light of indications of the range of social, health and environmental consequences and costs of overcrowding, it is a concern that in 2013, 62.9% of the South African population was estimated to be living in urban areas, and that in the coming years this figure is expected to increase by 2.4% annually [34]. A failure to match the associated demand for housing, with appropriate housing delivery programmes, as well as a failure to address the existing housing backlog, may lead to sustained or exacerbated overcrowding in urban settings. In turn, persistent overcrowding may aggravate the concomitant, avoidable burden of social, health and economic consequences for the country.

This study has some limitations inherent to a cross-sectional epidemiological study design. Firstly, the study cannot provide any evidence of causality. Secondly, there may be information bias at the respondent level since overcrowding and health outcomes were constructed on the respondent’s ability to remember household events and activities accurately. Thirdly, both acute gastrointestinal symptoms and respiratory symptoms are linked to poor hygiene, which was not assessed in this study. Fourthly, a 2-week recall period of the prevalence of respiratory and acute gastrointestinal symptoms over 12-month prevalence was used in this study, as previous studies had shown that this definition is less susceptible to recall bias [35, 36]. Lastly, the unwillingness of the respondents to provide honest answers or giving socially desirable responses should be taken into account in the interpretation of the results.

## Conclusion

In this study, conducted in two settings of poverty in the city of Johannesburg, South Africa, 57.6% of dwellings were found to be overcrowded in terms of the UN-HABITAT definition. Overcrowding was associated with elevated levels of acute respiratory and gastrointestinal symptoms, as well as fever/chills. Action to reduce crowded living conditions would support Sustainable Development Goals 3 (health), 10 (reduced inequalities) and 11 (sustainable cities and communities), while a failure to address the prevailing levels of overcrowding maybe posing a significant drain on the South African economy, in terms of the concomitant health and social consequences.

## Supplementary information

**Additional file 1:** **Table S1.** Univariate analyses of the degree of dwelling overcrowding by UN HABITAT definition, acute respiratory and gastrointestinal symptoms, fever/chills among the study participants in the study sites. Multiple regression analysis of dwelling overcrowding, acute respiratory and acute gastrointestinal symptoms, fever/chills. **Table S2.** Multiple regression analysis of dwelling overcrowding and wet cough among the participants. **Table S3.** Multiple regression analysis of dwelling overcrowding and dry cough among the participants. **Table S4.** Multiple regression analysis of dwelling overcrowding and runny/blocked nose among the participants. **Table S5.** Multiple regression analysis of dwelling overcrowding and diarrhea among the participants. **Table S6.** Multiple regression analysis of dwelling overcrowding and vomiting among the participants. Multiple regression analysis of dwelling overcrowding and fever/chills among the participants.

## Data Availability

We did not receive research ethics approval to share raw data field data publicly. The data belongs to the South African Medical Research Council (SAMRC). The raw dataset analyzed in the current study are available from the SAMRC on reasonable request.

## Abbreviations

CI: Confidence Intervals
LRA: Logistic Regression Analysis
OR: Odd ratio
R: South African Rand
SAMRC: South African Medical Research Council
UN-HABITAT: United Nations-HABITAT
US$: US Dollars
