# Supplementary material for: Overcrowding and health in two impoverished suburbs of Johannesburg, South Africa
Source: BMC Public Health. 2019 Oct 24;19:1358. doi: 10.1186/s12889-019-7665-5 (PMC6813127; doi:10.1186/s12889-019-7665-5)
Supplement: Supplementary file 1 — Additional file 1: Table S1. Univariate analyses of the degree of dwelling overcrowding by UN HABITAT definition, acute respiratory and gastrointestinal symptoms, fever/chills among the study participants in the study sites. Multiple regression analysis of dwelling overcrowding, acute respiratory and acute gastrointestinal symptoms, fever/chills. Table S2. Multiple regression analysis of dwelling overcrowding and wet cough among the participants. Table S3. Multiple regression analysis of dwelling overcrowding and dry cough among the participants. Table S4. Multiple regression analysis of dwelling overcrowding and runny/blocked nose among the participants. Table S5. Multiple regression analysis of dwelling overcrowding and diarrhea among the participants. Table S6. Multiple regression analysis of dwelling overcrowding and vomiting among the participants. Multiple regression analysis of dwelling overcrowding and fever/chills among the participants. [file 12889_2019_7665_MOESM1_ESM.docx]

**Additional file 1 Table S1: Univariate analyses of the degree of dwelling overcrowding by UN HABITAT definition, acute respiratory and gastrointestinal symptoms, fever/chills among the study participants in the study sites**

|  | ***Acute respiratory symptoms*** | | | | | | | | | | |
| --- | --- | --- | --- | --- | --- | --- | --- | --- | --- | --- | --- |
|  | **Wet cough** | | | | **Dry Cough** | | | | **Runny/blocked nose** | | |
|  | **Crude OR** | **(95% CI)** | | **p-value** | **Crude OR** | **(95% CI)** | **p-value** | | **Crude OR** | **(95% CI)** | **p-value** |
| **Dwelling overcrowding** |  |  | |  |  |  |  | |  |  |  |
| Not overcrowded | 1 | 1 | | 1 | 1 | 1 | 1 | | 1 | 1 | 1 |
| Moderately overcrowded | 2.86 | 2.12 – 3.84 | | <0.001 | 2.47 | 1.85 – 3.31 | <0.001 | | 2.29 | 1.74 – 2.86 | <0.001 |
| Extremely overcrowded | 3.57 | 2.35 – 5.41 | | < 0.001 | 3.76 | 2.56 – 5.60 | <0.001 | | 3.07 | 2.15 – 4.39 | <0.001 |
|  |  |  | |  |  |  | | |  |  |  |
|  | **Acute gastrointestinal symptoms** | | | | | | | | | | |
|  | **Diarrhoea** | | | | | **Vomiting** | | | | | |
|  | **Crude OR** | **(95% CI)** | | **p-value** | | **Crude OR** | | | **(95% CI)** | | **p-value** |
| **Dwelling overcrowding** |  |  | |  | |  | | |  | |  |
| Not overcrowded | 1 | 1 | | 1 | | 1 | | | 1 | | 1 |
| Moderately overcrowded | 1.20 | 0.73 – 1.98 | | 0.48 | | 0.87 | | | 0.55 – 1.36 | | 0.540 |
| Extremely overcrowded | 2.13 | 1.07 – 4.28 | | 0.03 | | 1.21 | | | 0.60 – 2.46 | | 0.591 |
|  |  |  | |  | |  | | |  | |  |
|  |  |  | |  | |  | | |  | |  |
|  | ***Fever/Chills*** | | | | | | | | | | |
|  | **Crude OR** | | **(95% CI)** | | | | | **p-value** | | | |
| **Dwelling overcrowding** |  | |  | | | | |  | | | |
| Not overcrowded | 1 | | 1 | | | | | 1 | | | |
| Moderately overcrowded | 2.42 | | 1.85 – 3.16 | | | | | < 0.001 | | | |
| Extremely overcrowded | 3.08 | | 2.09 – 4.53 | | | | | <0.001 | | | |

|  | ***Acute respiratory symptoms*** | | | | | | | | | |
| --- | --- | --- | --- | --- | --- | --- | --- | --- | --- | --- |
|  | **Wet cough^a^** | | | **Dry Cough^b^** | | | | **Runny/blocked nose^c^** | | |
|  | **Adjusted OR** | **(95% CI)** | **p-value** | **Adjusted OR** | **(95% CI)** | **p-value** | | **Adjusted OR** | **(95% CI)** | **p-value** |
| **Dwelling overcrowding** |  |  |  |  |  |  | |  |  |  |
| Not overcrowded | 1 | 1 | 1 | 1 | 1 | 1 | | 1 | 1 | 1 |
| Moderately overcrowded | **1.87** | **1.32 – 2.63** | **<0.001** | **1.54** | **1.11 – 2.11** | **0.009** | | **1.34** | **1.01 – 1.77** | **0.046** |
| Extremely overcrowded | **1.96** | **1.19 – 3.22** | **0.008** | **1.94** | **1.19 – 3.16** | **0.008** | | **1.59** | **1.05 – 2.41** | **0.028** |
|  |  |  |  |  |  | | |  |  |  |
|  | ***Acute gastrointestinal symptoms*** | | | | | | | | | |
|  | **Diarrhoea^d^** | | | | **Vomiting^e^** | | | | | |
|  | **Adjusted OR** | **(95% CI)** | **p-value** | | **Adjusted OR** | | | **(95% CI)** | | **p-value** |
| **Dwelling overcrowding** |  |  |  | |  | | |  | |  |
| Not overcrowded | 1 | 1 | 1 | | 1 | | | 1 | | 1 |
| Moderately overcrowded | 1.19 | 0.68 – 2.08 | 0.42 | | 0.90 | | | 0.52 – 1.55 | | 0.700 |
| Extremely overcrowded | **2.57** | **1.15 – 5.75** | **0.022** | | 1.49 | | | 0.65 – 3.21 | | 0.361 |
|  |  |  |  | |  | | |  | |  |
|  |  |  |  | |  | | |  | |  |
|  | ***Fever/Chills^f^*** | | | | | | | | | |
|  | **Adjusted OR** | | **(95% CI)** | | | | **p-value** | | | |
| **Dwelling overcrowding** |  | |  | | | |  | | | |
| Not overcrowded | 1 | | 1 | | | | 1 | | | |
| Moderately overcrowded | **1.49** | | **1.08 – 2.05** | | | | **0.015** | | | |
| Extremely overcrowded | **1.60** | | **1.01 – 2.52** | | | | **0.044** | | | |

^a-f^: Models adjusted for sex, study area, head of the household education level, whether any member of the household smoke at home, household total monthly income, having children under the age of five in the household, and period of residence in the current dwelling

**Table S2: Multiple regression analysis of dwelling overcrowding and wet cough among the participants**

| **Wet cough** | **Odds ratio** | **95 % CI** | **P-value** |
| --- | --- | --- | --- |
| ***Sex*** |  |  |  |
| Female | 1 | 1 | 1 |
| Male | 1.20144 | .9068403 1.591744 | 0.201 |
| Area |  |  |  |
| Braamfischerville | 1 | 1 | 1 |
| Riverlea | 1.007767 | .5824505 1.743656 | 0.978 |
| ***Population group*** |  |  |  |
| Black | 1 | 1 | 1 |
| Coloured | 1.117713 | .653123 1.912783 | 0.684 |
| ***Children under the age of 5 at home*** |  |  |  |
| *No* | 1 | 1 | 1 |
| *Yes* | **4.879651** | **3.451418 6.898901** | **0.000** |
| ***Any member of the household smoke at home*** |  |  |  |
| No | 1 | 1 | 1 |
| Yes | **3.046566** | **2.300439 4.034692** | **0.000** |
| ***Period of residence in the area*** |  |  |  |
| < 10 years | 1 | 1 | 1 |
| > 10 years | 1.230098 | .9271645 1.632009 | 0.151 |
| ***Level of education (head of household)*** |  |  |  |
| None | 1 | 1 | 1 |
| Primary | **.5640214** | **.3412105 .9323282** | **0.026** |
| Secondary | **.4557652** | **.2853819 .7278734** | **0.001** |
| Tertiary | .8510785 | .4870199 1.487279 | 0.571 |
| ***Household total monthly income*** |  |  |  |
| No income | 1 | 1 | 1 |
| R1001 – R5000 | .9226285 | .6613441 1.287141 | 0.635 |
| R5001 – R 10000 | 1.186336 | .7523659 1.870623 | 0.462 |
| > R10000 | .560834 | .3079859 1.021263 | 0.059 |
|  |  |  |  |

**Table S3: Multiple regression analysis of dwelling overcrowding and dry cough among the participants**

| **Dry cough** | **Odds ratio** | **95 % CI** | **P-value** |
| --- | --- | --- | --- |
| ***Sex*** |  |  |  |
| Female | 1 | 1 | 1 |
| Male | 1.169817 | .874459 1.564937 | 0.290 |
| Area |  |  |  |
| Braamfischerville | 1 | 1 | 1 |
| Riverlea | 1.392484 | .7925502 2.446548 | 0.249 |
| ***Population group*** |  |  |  |
| Black | 1 | 1 | 1 |
| Coloured | 1.05534 | .6040912 1.843666 | 0.850 |
| ***Children under the age of 5 at home*** |  |  |  |
| *No* | 1 | 1 | 1 |
| *Yes* | **6.490475** | **4.588541 9.180753** | **0.000** |
| ***Any member of the household smoke at home*** |  |  |  |
| No | 1 | 1 | 1 |
| Yes | **2.816794** | **2.14014 3.707388** | **0.000** |
| ***Period of residence in the area*** |  |  |  |
| < 10 years | 1 | 1 | 1 |
| > 10 years | 1.25981 | .9191733 1.726684 | 0.151 |
| ***Level of education (head of household)*** |  |  |  |
| None | 1 | 1 | 1 |
| Primary | .8556767 | .504724 1.450659 | 0.562 |
| Secondary | .7028449 | .4234681 1.166536 | 0.172 |
| Tertiary | .7226168 | .3706571 1.408782 | 0.340 |
| ***Household total monthly income*** |  |  |  |
| No income | 1 | 1 | 1 |
| R1001 – R5000 | .916813 | .6503999 1.292353 | 0.620 |
| R5001 – R 10000 | 1.176011 | .7131442 1.939302 | 0.525 |
| > R10000 | .9804907 | .5635769 1.705822 | 0.944 |
|  |  |  |  |

**Table S4: Multiple regression analysis of dwelling overcrowding and runny/blocked nose among the participants**

| **Runny/blocked nose** | **Odds ratio** | **95 % CI** | **P-value** |
| --- | --- | --- | --- |
| ***Sex*** |  |  |  |
| Female | 1 | 1 | 1 |
| Male | .9432128 | .7376888 1.205997 | 0.641 |
| Area |  |  |  |
| Braamfischerville | 1 | 1 | 1 |
| Riverlea | 1.08782 | .6748097 1.75361 | 0.729 |
| ***Population group*** |  |  |  |
| Black | 1 | 1 | 1 |
| Coloured | 1.189742 | .7514507 1.883671 | 0.458 |
| ***Children under the age of 5 at home*** |  |  |  |
| *No* | 1 | 1 | 1 |
| *Yes* | **5.875143** | **4.298368 8.030326** | **0.000** |
| ***Any member of the household smoke at home*** |  |  |  |
| No | 1 | 1 | 1 |
| Yes | **3.625663** | **2.808236 4.68103** | **0.000** |
| ***Period of residence in the area*** |  |  |  |
| < 10 years | 1 | 1 | 1 |
| > 10 years | **1.361516** | **1.057913 1.752248** | **0.017** |
| ***Level of education (head of household)*** |  |  |  |
| None | 1 | 1 | 1 |
| Primary | .8344232 | .50358 1.382625 | 0.482 |
| Secondary | .8202597 | .5089384 1.322019 | 0.415 |
| Tertiary | .9434359 | .5002835 1.779134 | 0.857 |
| ***Household total monthly income*** |  |  |  |
| No income | 1 | 1 | 1 |
| R1001 – R5000 | .7531663 | .5572692 1.017927 | 0.065 |
| R5001 – R 10000 | 1.137402 | .7610645 1.699833 | 0.530 |
| > R10000 | .6736444 | .4010972 1.131388 | 0.135 |
|  |  |  |  |

**Table S5: Multiple regression analysis of dwelling overcrowding and diarrhea among the participants**

| **Diarrhea** | **Odds ratio** | **95 % CI** | **P-value** |
| --- | --- | --- | --- |
| ***Sex*** |  |  |  |
| Female | 1 | 1 | 1 |
| Male | 1.091632 | .6547307 1.820078 | 0.736 |
| Area |  |  |  |
| Braamfischerville | 1 | 1 | 1 |
| Riverlea | 1.53905 | .5929695 3.994598 | 0.375 |
| ***Population group*** |  |  |  |
| Black | 1 | 1 | 1 |
| Coloured | .5997331 | .243016 1.480066 | 0.267 |
| ***Children under the age of 5 at home*** |  |  |  |
| *No* | 1 | 1 | 1 |
| *Yes* | .8921037 | .5163954 1.541162 | 0.682 |
| ***Any member of the household smoke at home*** |  |  |  |
| No | 1 | 1 | 1 |
| Yes | **3.486174** | **2.113756 5.749674** | **0.000** |
| ***Period of residence in the area*** |  |  |  |
| < 10 years | 1 | 1 | 1 |
| > 10 years | **1.684246** | **1.037618 2.733844** | **0.035** |
| ***Level of education (head of household)*** |  |  |  |
| None | 1 | 1 | 1 |
| Primary | 7427881 | .2637554 2.09184 | 0.573 |
| Secondary | .7146512 | .2756568 1.852762 | 0.489 |
| Tertiary | .976576 | .3069503 3.10702 | 0.968 |
| ***Household total monthly income*** |  |  |  |
| No income | 1 | 1 | 1 |
| R1001 – R5000 | 1.14517 | .5894462 2.224825 | 0.689 |
| R5001 – R 10000 | **2.490549** | **1.122957 5.523661** | **0.025** |
| > R10000 | 1.662535 | .7019602 3.937576 | 0.248 |
|  |  |  |  |

**Table S6: Multiple regression analysis of dwelling overcrowding and vomiting among the participants**

| **Vomiting** | **Odds ratio** | **95 % CI** | **P-value** |
| --- | --- | --- | --- |
| *Sex* |  |  |  |
| Female | 1 | 1 | 1 |
| Male | 1.204849 | .7504186 1.934468 | 0.440 |
| Area |  |  |  |
| Braamfischerville | 1 | 1 | 1 |
| Riverlea | .5042892 | .2445754 1.039792 | 0.064 |
| ***Population group*** |  |  |  |
| Black | 1 | 1 | 1 |
| Coloured | 1.925253 | .9082394 4.081081 | 0.087 |
| ***Children under the age of 5 at home*** |  |  |  |
| *No* | 1 | 1 | 1 |
| *Yes* | 1.049715 | .6284873 1.75326 | 0.853 |
| ***Any member of the household smoke at home*** |  |  |  |
| No | 1 | 1 | 1 |
| Yes | **6.438798** | **3.715197 11.15906** | **0.000** |
| ***Period of residence in the area*** |  |  |  |
| < 10 years | 1 | 1 | 1 |
| > 10 years | 1.096688 | 6810664 1.765943 | 0.704 |
| ***Level of education (head of household)*** |  |  |  |
| None | 1 | 1 | 1 |
| Primary | .4988343 | .2186428 1.138093 | 0.098 |
| Secondary | **.4177079** | **.198351 .8796525** | **0.022** |
| Tertiary | .3822879 | .1331692 1.097431 | 0.074 |
| ***Household total monthly income*** |  |  |  |
| No income | 1 | 1 | 1 |
| R1001 – R5000 | 1.245162 | .7129501 2.174666 | 0.440 |
| R5001 – R 10000 | 1.437102 | .6299547 3.278428 | 0.388 |
| > R10000 | **2.482852** | **1.189805 5.181143** | **0.015** |
|  |  |  |  |

**Table S6: Multiple regression analysis of dwelling overcrowding and fever/chills among the participants**

| **Fever/Chills** | **Odds ratio** | **95 % CI** | **P-value** |
| --- | --- | --- | --- |
| ***Sex*** |  |  |  |
| Female | 1 | 1 | 1 |
| Male | 1.204849 | .7504186 1.934468 | 0.440 |
| Area |  |  |  |
| Braamfischerville | 1 | 1 | 1 |
| Riverlea | .5042892 | .2445754 1.039792 | 0.064 |
| ***Population group*** |  |  |  |
| Black | 1 | 1 | 1 |
| Coloured | 1.925253 | .9082394 4.081081 | 0.087 |
| ***Children under the age of 5 at home*** |  |  |  |
| *No* | 1 | 1 | 1 |
| *Yes* | 1.049715 | .6284873 1.75326 | 0.853 |
| ***Any member of the household smoke at home*** |  |  |  |
| No | 1 | 1 | 1 |
| Yes | **6.438798** | **3.715197 11.15906** | **0.000** |
| ***Period of residence in the area*** |  |  |  |
| < 10 years | 1 | 1 | 1 |
| > 10 years | 1.096688 | .6810664 1.765943 | 0.704 |
| ***Level of education (head of household)*** |  |  |  |
| None | 1 | 1 | 1 |
| Primary | .4988343 | .2186428 1.138093 | 0.098 |
| Secondary | **.4177079** | **.198351 .8796525** | **0.022** |
| Tertiary | .3822879 | .1331692 1.097431 | 0.074 |
| ***Household total monthly income*** |  |  |  |
| No income | 1 | 1 | 1 |
| R1001 – R5000 | 1.245162 | .7129501 2.174666 | 0.440 |
| R5001 – R 10000 | 1.437102 | .6299547 3.278428 | 0.388 |
| > R10000 | **2.482852** | **1.189805 5.181143** | **0.015** |
|  |  |  |  |
